# Non-Alcoholic Fatty Liver Disease and Coronary Artery Disease: A Bidirectional Association Based on Endothelial Dysfunction

**DOI:** 10.3390/ijms251910595

**Published:** 2024-10-01

**Authors:** Nikolaos Ktenopoulos, Marios Sagris, Maria Gerogianni, Konstantinos Pamporis, Anastasios Apostolos, Konstantinos Balampanis, Konstantinos Tsioufis, Konstantinos Toutouzas, Dimitris Tousoulis

**Affiliations:** 1 First Department of Cardiology, ‘Hippokration’ General Hospital, School of Medicine, National and Kapodistrian University of Athens, 11527 Athens, Greece; masagris1919@gmail.com (M.S.); anastasisapostolos@gmail.com (A.A.); ktsioufis@gmail.com (K.T.); ktoutouz@gmail.com (K.T.); drtousoulis@hotmail.com (D.T.); 2 Endocrine Unit, 2nd Propaedeutic Department of Internal Medicine, School of Medicine, Research Institute and Diabetes Center, Attikon University Hospital, National and Kapodistrian University of Athens, 12641 Athens, Greece; gerogianni.e.maria@gmail.com; 3 Second Department of Internal Medicine, Attikon University Hospital, Medical School, National and Kapodistrian University of Athens, 12462 Athens, Greece; kostasbalabanis@gmail.com; 4 Department of Hygiene, Social-Preventive Medicine & Medical Statistics, Medical School, Aristotle University of Thessaloniki, University Campus, 54124 Thessaloniki, Greece; konstantinospab@gmail.com

**Keywords:** non-alcoholic fatty liver disease, NALFD, endothelial dysfunction, coronary artery disease, CAD, prevention, treatment, genetics

## Abstract

Non-alcoholic fatty liver disease (NAFLD) is the most common cause of chronic liver disease and is regarded as a liver manifestation of metabolic syndrome. It is linked to insulin resistance, obesity, and diabetes mellitus, all of which increase the risk of cardiovascular complications. Endothelial dysfunction (EnD) constitutes the main driver in the progression of atherosclerosis and coronary artery disease (CAD). Several pathophysiological alterations and molecular mechanisms are involved in the development of EnD in patients with NAFLD. Our aim is to examine the association of NAFLD and CAD with the parallel assessment of EnD, discussing the pathophysiological mechanisms and the genetic background that underpin this relationship. This review delves into the management of the condition, exploring potential clinical implications and available medical treatment options to facilitate the deployment of optimal treatment strategies for these patients.

## 1. Introduction

Non-alcoholic fatty liver disease (NAFLD) is a medical term that was first described by Ludwig et al. [1] in 1980 encompassing individuals with fatty liver disease without excess alcohol consumption, characterized by <20 gr/day for females and <30 gr/day for males. As of 2002, the term NAFLD covers a wide spectrum of presentations and stages, and specifically it encompasses simple hepatic steatosis (stage 1—fat deposition in the liver), non-alcoholic steatohepatitis (NASH–stage 2—inflammation), fibrosis (stage 3—the liver along with its surrounding blood vessels develop scar tissue as a result of chronic inflammation, although the liver itself continues to function effectively), and cirrhosis (stage 4—the most severe stage, which follows years of inflammation and causes the liver to shrink and become scarred; this is irreversible and can result in liver failure or even cancer). It mainly constitutes an asymptomatic condition, making it difficult to diagnose in early stages [2]. NAFLD typically coincides with other chronic metabolic conditions such as obesity and diabetes mellitus (DM), which increase the risk of cardiovascular diseases, mainly inducing the progression of the atherosclerotic disease [3,4]. Given the link between NAFLD and obesity, it is quite clear that NAFLD has become the most prominent hepatic condition with the prevalence of NAFLD in the United States fluctuating from 5.5% to 11% [5,6].

As is well documented, NAFLD diagnosis requires imaging or histological evidence of liver steatosis, and the absence of further causes of fat accumulation in the liver, such as excess alcohol consumption, long-term use of medications that cause steatosis, such as the use of corticosteroids or amiodarone, the presence of hepatitis C virus infection, congenital disorders, malnutrition, or hepatolenticular degeneration [3]. Even once alternative causes of steatosis are ruled out, NASH represents a tremendously diverse group of patients, and a variety of variables have been hypothesized to hasten the disease’s progression. Alcohol intake, for example, is one of the most perplexing elements in the evolution of NAFLD, since it is difficult to quantify the extent to which it affects it [3].

Metabolic syndrome is a term that includes central obesity, hypertension, insulin resistance, and dyslipidemia, and is closely linked to an increased risk of cardiovascular disease (CVD) and type 2 diabetes mellitus (T2DM) [7]. Alongside, its associated co-morbidities constitute factors that contribute to NAFLD progression, and they co-exist in 36–67% of patients [8,9]. Among them, male sex, hyperuricemia, and metabolic syndrome’s components, such as obesity, arterial hypertension, or dyslipidemia, constitute some of the well-established NAFLD induction factors [10]. There is also evidence of the pathophysiological link between NAFLD and endothelial dysfunction (EnD), which is a precursor stage of atherosclerosis. Inflammation, oxidative stress, and apoptosis are all associated with the development of NAFLD [11]. NAFLD not only triggers atherosclerosis, but it also increases the risk of developing coronary artery disease (CAD), valvular heart disease, heart failure, and stroke [12].

In this narrative review, we summarize the current evidence regarding the pathophysiology of EnD in NAFLD as a main driver in the progression of atherosclerosis. We also examine the association of NAFLD and CAD with a parallel assessment of endothelial function, discussing the pathophysiological mechanisms that underpin this relationship.

## 2. Epidemiology and the Consensus of NAFLD Terminology

Being a worldwide pandemic, NAFLD comprises the most common [6] and most frequently diagnosed [8] chronic liver disease and is expected to become the primary indication for hepatic transplantation [13]. It has been determined that 20 to 30% of the general population is affected, with 64 million patients in the United States of America (USA) and 52 million in European countries, with a linearly increasing trend. Notably, most NAFLD patients, around 91%, remain at the early stages of the disease, given its slow progressive nature [14]. With an economic burden in the United States for NAFLD at $103 billion and in Europe countries at €35 billion annually, it is relatively simple to compare and associate it with DM and cardiovascular disease (CVD), which are the leading economic burden components in the USA [15]. Of note, these costs were the highest among patients aged between 45 and 65 years old [14], which constitutes an extra problem for modern healthcare systems and public health, considering the importance of that age group in society.

In 2016 Younossi et al. [8] conducted a meta-analysis referring to the global epidemiology of NAFLD, including 8,515,431 patients from a total of 85 studies. They also found that NAFLD detected by imaging had a prevalence of roughly 25.2% (95% CI: 22.10–28.65), with South America (30.45%; 95% CI: 22.74–39.44) and Middle East (31.79%; 95% CI: 13.48–58.23) presenting the highest percentages, while Africa presented the lowest (13.48%; 95% CI: 5.69–28.69). In a more recent analysis, Wang et al. showed that internationally NAFLD burdens have expanded since 1990, with estimated annual percentage changes of 0.77 [95% CI (0.69, 0.85)] [16]. Of note, disparities in the available diagnostic means across different geographic regions might underestimate its prevalence in certain areas. The incidence of specific comorbidities associated with metabolic syndrome were calculated and associated with NAFLD. Obesity presented in 51.3% of the cases, hyperlipidemia in 69.1%, hypertension in 39.3%, T2DM in 22.5%, and complete metabolic syndrome in 42.5%. The estimated overall mortality among NAFLD patients was found to be elevated with an adjusted hazard ratio of 1.04 (1.03–1.04) [8]. Interestingly, there has also been reported an increasing risk of mortality with worsening NAFLD disease histological stage [17].

Furthermore, CVD and NAFLD appear to be closely related, since individuals with NAFLD present with an elevated risk of CVD events such as myocardial infarction requiring revascularization or stroke [18]. Multiple common risk factors have been identified for both NAFLD and CVD, such as increased body mass index (BMI), T2DM, dyslipidemia, or a lack of physical activity, suggesting the existence of shared pathophysiological pathways [19]. Additionally, CVD is the leading cause of death in patients with NAFLD, highlighting the necessity of detecting and managing CVD risk in these patients [20].

## 3. Metabolic-Associated Fatty Liver Disease (MAFLD) and Metabolic Dysfunction-Associated Steatotic Liver Disease (MASLD)

In 2019 Eslam et al. [21], on behalf of an international consensus panel, tried to update the NAFLD term since it does not consider the current knowledge. The expert group suggested the term MAFLD as a valid alternative. They mentioned the fact that the previous term includes a wide spectrum of stages and phenotypes, but it sometimes fails to reflect several aspects: First, the previous term defines a diagnosis by exclusion of certain entities such as hepatitis C or increased alcohol consumption, while current knowledge suggests that these conditions may co-exist and may also act synergistically in liver disease progression [22]. Additionally, alcohol consumption determination for NAFLD diagnosis is under debate, since it is subjective, and even though low amounts are considered normal, it adds stigma to its name, affecting patients. As a result, the co-existence of non-alcoholic and alcoholic liver disease is misinterpreted.

Moreover, the separation of patients into NASH and no-NASH stages in clinical practice is problematic, since it does not take into consideration fibrosis, which is a strong determinant to adverse events [23]. The heterogeneous nature of fatty liver diseases highlights the need for a deeper understanding of their pathophysiology and the development of tailored therapeutic strategies. The term NAFLD often leads to a ‘single-condition approach’, failing to capture the diverse risk profiles and treatment options required for effective management. To address these challenges, an international consensus committee proposed the term MAFLD, which more accurately reflects the current understanding of fatty liver disease and its associations.

This shift in terminology is crucial, given the rising prevalence and global impact of NAFLD, which poses a significant burden on healthcare systems. Unlike NAFLD, the term MAFLD emphasizes the importance of identifying obesity and metabolic dysfunctions as high-risk factors, offering a more comprehensive diagnostic approach. Moreover, the MAFLD definition excludes alcohol consumption and other concurrent liver diseases from influencing the diagnosis, thereby reducing the stigma and providing a clearer path for personalized treatment strategies. By adopting MAFLD, the classification and understanding of fatty liver diseases can be improved, promoting more effective management and therapeutic interventions [24].

Finally, in 2023, several national liver associations introduced an additional term, ‘Metabolic Dysfunction-Associated Steatotic Liver Disease’ (MASLD), to further refine diagnosis. MASLD is defined as liver steatosis in the presence of at least one cardiometabolic risk factor, encompassing all liver diseases with pathological fat accumulation [25]. Although the NAFLD, MAFLD, and MASLD terminologies differ, their commonalities lie in addressing the cardiometabolic factors associated with liver disease. Given the ongoing transition and incomplete adoption of the MAFLD and MASLD terms within the scientific community, we have chosen to retain the term NAFLD in this review to ensure consistency and clarity [24,25,26].

## 4. NAFLD and Coronary Artery Disease

Fatty liver is highly correlated with an elevated risk of CAD, and at the same time, CAD is a leading cause of mortality in those with NAFLD. This phenomenon may also be elucidated by the observation that the development of fat in the liver frequently corresponds with the accumulation of fat in the heart and an elevated resistance to insulin in these individuals. Nevertheless, although there is a significant connection between metabolic syndrome and CAD, research has demonstrated that certain components of metabolic syndrome, such as DM and hypertension, are more powerful individual predictors of CAD than metabolic syndrome as a whole. Additionally, the association between NAFLD and CAD remains unaffected by other demographic and metabolic factors [27].

## 5. Shared Risk Factors of CAD and NAFLD

At present, NAFLD is recognized as a complex metabolic disorder arising from intricate interactions among genetic susceptibility, host metabolic disturbances, and environmental factors. It is closely associated with major cardiovascular risk factors such as hypertension, dyslipidemia, obesity, and DM mainly due to insulin resistance [28]. As is widely acknowledged, the presence of the aforementioned risk factors not only exacerbates the risk of CAD progression but also contributes to the occurrence of major adverse cardiovascular events (MACE). Consequently, it appears that NAFLD and CAD share common risk factors and pathophysiological pathways, establishing a bidirectional and potentially lethal relationship. Age and gender are among the non-modifiable risk factors, with males exhibiting higher prevalence [29]. However, women display a consistent increase in prevalence with age, while men maintain a similar prevalence across all age groups. Physical inactivity emerges as a significant contributing factor, promoting excessive calorie intake that surpasses the expansion and storage capacity of adipose tissue [30]. This leads to elevated serum levels of free fatty acids and the formation of ectopic fat deposits in the liver and other organs. Patients with NAFLD develop progressing insulin resistance due to accumulation of fatty acid in liver which leads to hyperglycemia, hyperinsulinemia, and potentially hyperlipidemia. NAFLD is also associated with dyslipidemia, given that lipid intake, synthesis, and regulation of metabolism are impaired in the livers of patients with NAFLD due to enlarged fat mass. There is a down-regulation of low-density lipoprotein (LDL)-receptors resulting in concomitant elevation of cholesterol, very-low density lipoprotein, and triglyceride levels [31]. The majority of individuals with NAFLD, often accompanied by hypertension, represent a significant risk group for the progression of CAD. The presence of hypoadiponectinemia in these patients contributes to impaired endothelium-dependent vasoreactivity, potentially serving as a causative factor for hypertension [31]. In conclusion, both conditions exhibit a relatively stable inflammatory state marked by increased levels of tumor necrosis factor-α, interleukin (IL)-6, monocyte chemoattractant protein-1, and C-reactive protein, along with dysfunction of the fibrinolytic system [28].

## 6. Pathophysiological Mechanisms and Association of CAD and NAFLD

Despite not being fully understood, the evidence enhances the suggestion for shared pathophysiological pathways between NAFLD and CAD [32]. The proposed mechanisms include: EnD with a disruption in nitric oxide production, elevated levels of homocysteine with an inflammatory mediator, and systemic inflammation with an increase in high sensitivity C-reactive protein and lipoprotein A. Lipid metabolism dysregulation with adiponectin, gut microbiota with endotoxins, and bile acids and environmental factors like insulin resistance and/or various genetic factors have also been described to contribute to the common pathophysiology between NAFLD and CAD.

All these eventually accelerate atherosclerosis and plaque formation within the vessels and contribute to the progression to CAD. Insulin resistance, irregular lipid profiles, oxidative stress, and inflammatory processes are pathogenic factors that can cause non-alcoholic steatohepatitis and exacerbate the course of cardiovascular disease [32]. Thus, it would be adequate to assume that different NASH phenotypes correspond to more serious impairment of the coronary arteries than liver steatosis itself [33]. 

**Figure 1:**
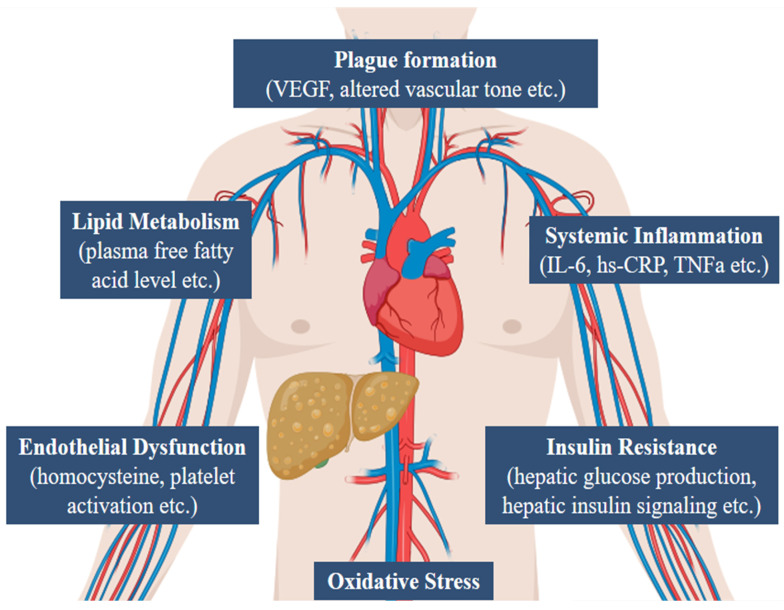
The multifactorial burden of NAFLD. Abbreviations: vascular endothelial growth factor (VEGF), interleukin 6 (IL-6), high-sensitivity C-reactive protein (hs-CRP), tumor necrosis factor alpha (TNFa).

Figure 1 summarizes the different pathophysiological mechanisms of NAFLD. 

## 7. The Role of the Endothelium in Atherosclerosis

Atherosclerosis is a cardiovascular condition represented by the accumulation of lipids, fibrous components, and calcification inside the major arteries. Endothelial activation, which triggers a chain reaction that contributes to the development of atheromatous plaques, eventually results in CVD, which is still the most prevalent cause of mortality worldwide [34].

Firstly, the endothelium through the production of nitric oxide forces the loosening of the smooth muscle of the blood vessels. Conditions like T2DM and hypertension both reduce endothelial nitric oxide release. Likewise, arteries with regenerated endothelium also present with impaired nitric oxide production [35]. These alterations facilitate vasospasm, clotting, macrophage entry, cell growth, and the inflammatory response that leads to atherosclerosis initiation. Endothelial cells also produce endothelium-derived contraction factor, which causes vasoconstriction, while aging, high blood pressure, and T2DM further enhance this process. This, the first step towards atherosclerosis is EnD through the creation of fatty streaks or plaque inflammation [35], while it serves as an independent risk factor for CAD initiation in NAFLD patients [36].

## 8. Endothelial Dysfunction and NAFLD

EnD is an initial phase in the formation of atherosclerosis and is thus also critical in the progression of cardiovascular disease. This pertains to the connection between oxidative stress triggered by superoxide, inflammation of the blood vessels stimulated by lipoproteins (such as apolipoprotein C3), and selective vascular insulin resistance. EnD is marked by an overall decrease in the amount of nitric oxide (NO), which is a substance that functions to protect the vessels and promotes their dilation. Increased concentrations of asymmetric dimethylarginine in the bloodstream, which acts as a natural inhibitor of nitric oxide synthase (NOS), can reduce the availability of NO. This reduction in NO levels may contribute to insufficient control of blood vessels’ regulation, as well as increased vascular perviousness and dysfunction of the platelets. EnD in the vascular structure of the liver and the intestines is very well-documented in cirrhosis [35], while it has also been observed in NAFLD. The main cause of elevated levels of asymmetric dimethylarginine in individuals with NAFLD is insulin resistance [37], since asymmetric dimethylarginine acts as a natural antagonist to nitric oxide synthase [38].

Additionally, high levels of homocysteine in the blood serum have been recognized, mostly because of alterations to methionine metabolism, which interfere with the processes by which homocysteine is synthesized and metabolized through the liver [39]. Hyperhomocysteinemia is linked to higher resistance in the intrahepatic vessels, through reduced replication of glutathione storage and nitric oxide production. As a result, the above mechanisms lead to disrupted levels of nitric oxide, a vital component of vascular relaxation [37]. Moreover, hyperhomocysteinemia results in oxidative stress which leads to intensified platelet activation [37].

Another component of normal vessel wall function is the undisrupted endothelium layer. Atherosclerosis is caused in part by injury to this layer, which is shown as an elevation in both endothelial microparticles, demonstrating injury to the endothelial layer, and endothelial progenitor cells, which reveal endothelial restoration [40]. In NAFLD, the amount of endothelial progenitor cells in the bloodstream is reduced and their ability to adherence is lessened [41]. Pastori et al. [42] studied 367 individuals, 281 of whom had NAFLD, evaluating endothelial function in this group using brachial artery flow-mediated dilation (FMD). They showed an inverse correlation between NAFLD and FMD, with FMD levels being significantly reduced in NAFLD patients (*p* < 0.001).

Similar data were published by Sapmaz et al. [43] in 2016, where 266 patients were studied, 176 of them with NAFLD. The NAFLD group appeared to have lower FMD rates (*p* = 0.008), indicating severe EnD. Moreover, a systematic review and meta-analysis in 2022 [44] including 22 studies with 2164 patients with NAFLD and 3322 patients in the control group, showed that the first group had lower FMD rates (SMD: −1.37, 95% CI −1.91 to −0.83, *p* < 0.001, I^2^: 98%). The study found that NAFLD individuals diagnosed using liver ultrasonography or liver biopsy had a considerably lower FMD compared to those diagnosed using a combination of techniques or other methods. However, there were no detected variations based on the selected cuff inflation threshold, the presence of a notable difference in obesity metrics between the groups, or the classification of the control group. In addition, this meta-analysis found that those with non-alcoholic steatohepatitis had a considerably lower FMD compared to those with pure steatosis (SMD: −0.81, 95% CI −1.51 to −0.31, *p* = 0.003, I^2^: 81%). Consequently, individuals with NAFLD witnessed a notable decrease in the FMD of the brachial artery, which is clear evidence of ED.

The main approach for identifying EnD is by the assessment of brachial artery FMD. Furthermore, another diagnostic technique is strain-gauge plethysmography, that demonstrates a substantial reduction in hypertensive individuals with hepatic steatosis compared to those without, making it less apparent than FMD [45].

## 9. Systemic Inflammation and NAFLD

Individuals with NAFLD typically have increased levels of inflammatory substances in their circulation, including interleukin 6, high sensitivity C-reactive protein, interleukin 1b, and tumor necrosis factor-a [37]. Systemic inflammation triggers EnD, modulates vascular tone, and intensifies accumulation of plaque in the vessels. The key findings in a study of patients with NAFLD corroborate these processes, since they revealed a substantial decrease in flow-mediated vasodilation compared to the control group (although BMI matching was not conducted) [46].

## 10. Plaque Formation/Instability and NAFLD

Individuals diagnosed with NAFLD may also have a greater risk of developing atherosclerotic CVD as a result of an imbalance in procoagulant substances. NAFLD patients often exhibit elevated levels of coagulation factors FVIII, FIX, FXI, and FXII in their bloodstream, along with increased levels of fibrinogen, von Willebrand factor, and plasminogen activator inhibitor-1, and reduced levels of antithrombin III and protein C. They may also experience altered blood concentrations of vascular endothelial growth factor (VEGF) that affect atherogenesis and plaque instability. Patients with NAFLD have increased levels of VEGF in their blood, indicating active angiogenesis and vascular remodeling. These changes are linked to the emergence and instability of plaques. Nevertheless, the existing evidence is inconclusive, and more study is required to determine the atherogenic impact of VEGF in NAFLD [37].

## 11. Platelet-Activating Factor as the Connecting Link between NAFLD and CAD

Recent evidence suggests that platelet-activating factor (PAF), a potent inflammatory lipid mediator, serves as a critical link between NAFLD and CAD by inducing EnD [47]. PAF is recognized for its role in promoting inflammation, platelet activation, and atherogenesis, all of which are key processes in the pathophysiology of both NAFLD and CAD [48]. Understanding the role of PAF in this complex interplay is crucial, as it provides insights into how NAFLD exacerbates cardiovascular risks through EnD. Furthermore, PAF has been established as a significant mediator of hepatotoxicity, capable of inducing inflammation and modifying liver function through PAF receptor antagonism [49]. This mediator’s role extends beyond liver injury, as it is implicated in the pathogenesis of acute liver failure and drug-induced liver injury (DILI), where inhibition of PAF has shown potential protective effects [50]. Furthermore, PAF plays an essential role in the progression of cardiovascular disease, specifically through EnD, by promoting atheromatous plaque formation and accelerating atherosclerosis. The therapeutic implications of targeting PAF are becoming increasingly relevant in managing NAFLD and its associated cardiovascular complications [51]. While some PAF inhibitors have been shown to attenuate inflammatory responses, rupatadine stands out as the only commercially available drug that inhibits PAF-induced platelet activation. This suggests that rupatadine and other PAF antagonists may offer promising treatment avenues for NAFLD patients with concurrent cardiovascular risks, but further research is needed [51].

## 12. Gut Microbiota and NAFLD

The gastrointestinal tract may be considered a potential source of systemic inflammatory alterations, which might have a significant impact on metabolic disorders including NAFLD and CVD. Gut dysbiosis is characterized by an imbalance of the intestinal barrier, leading to an elevated permeability of the mucosal barrier. As a result, certain substances produced by intestinal microbes, known as pathogen-associated molecular patterns (PAMPs), such as lipopolysaccharides or peptidoglycans, as well as substances released from impaired enterocytes, known as damage-associated molecular patterns (DAMPs), enter the bloodstream and trigger different cellular signaling pathways. This leads to a systemic inflammatory response that is associated with an imbalance of gut bacteria. A disruption in the gut microbiota may trigger inflammation, which, in turn, links the gut microbiota to the development of CVD. Specifically, individuals with CAD and NAFLD had notable changes in their intestinal microbiota, characterized by a reduction in the abundance of Colinsella and Parabacterioides, which persisted over time [12]. This observation may perhaps account for the poorer clinical outcome and disease progression observed in these individuals in comparison to those with CAD but without NAFLD.

## 13. Lipid Metabolism Dysregulation and NAFLD

The liver is essential for lipid metabolism through processes such as lipogenesis, lipid breakdown, and serum lipoprotein absorption and release. Non-alcoholic fatty liver disease (NAFLD) disrupts the composition of lipids in the circulatory system, leading to abnormally high levels of triglyceride (TG), very low-density lipoprotein (VLDL), LDL, and abnormally low levels of high-density lipoprotein (HDL) [52]. Individuals who are obese, have T2DM, and have metabolic syndrome have excessive VLDL production as a result of elevated levels of free fatty acids in the bloodstream and increased levels of fat in the liver. Hepatic lobular inflammation has been associated with elevated blood concentrations of VLDL and LDL, regardless of the presence of steatosis. Additional examination of lipoprotein subclasses indicates that individuals with NASH exhibit a notable reduction in the size and peak diameter of LDL particles, along with an increased concentration of LDL particles, elevated levels of LDL (IVb), and decreased levels of HDL(IIb) [52]. These findings suggest a potential mechanism for an elevated risk of CVD in individuals with more severe NAFLD. The modified structure of blood lipoproteins is believed to contribute to the heightened risk for CVD. Nevertheless, it is important to acknowledge that the new findings do not indicate an association between elevated HDL levels and cardiovascular protection [53].

## 14. Insulin Resistance and NAFLD

NAFLD pathophysiology is very closely associated with insulin resistance, which is a risk factor for CVD. Obesity and excess free fatty acids not only lead to muscle insulin resistance but also induce hepatic insulin resistance and reduce insulin clearance. The mechanism by which NAFLD is associated with hepatic insulin resistance is believed to be due to increased hepatic diacylglycerol, which activates protein kinase C, resulting in decreased insulin signaling [33]. Fatty acid accumulation in the liver, primarily from adipose tissue lipolysis, also leads to a suppression of endogenous liver glucose production, further stimulating insulin resistance. Saturated fatty acids produce intrahepatic oxidative stress, which further impairs hepatic insulin signaling. Importantly, the relationship between NAFLD and CVD appears to be in addition to the risk conferred by DM, as the prevalence of CVD in patients with DM and NAFLD is increased compared with the risk in individuals with DM without NAFLD (OR: 1.6; 95% CI: 1.2 to 1.8).

## 15. Studies Associating CAD and NAFLD

Recent research has been exploring the pathophysiological pathways linking NAFLD and CAD. Vilar et al. conducted a study involving 244 Brazilian patients, where they found that 63.5% had CAD and 42.2% had NAFLD. Remarkably, 43.9% of the CAD patients also had NAFLD, demonstrating a strong association between the two conditions. A regression analysis further confirmed this link, showing that NAFLD was significantly correlated with CAD, with insulin resistance and an increased body mass index being prominent factors [54]. Similarly, Ampuero et al. investigated the impact of NAFLD on subclinical atherosclerosis and cardiovascular risk. By examining carotid intima-media thickness and the presence of carotid plaques in 612 patients, they observed that 80.4% of NAFLD patients had CAD compared to 60.7% without NAFLD (*p* < 0.001), indicating that NAFLD is associated with a higher risk of CAD and subclinical atherosclerosis [55]. 

In a systematic review and meta-analysis, Montovani et al. analyzed data from multiple studies and concluded that NAFLD significantly increases the risk of cardiovascular events, independent of other risk factors such as age, sex, adiposity measures, and DM [56]. Wild et al., in a retrospective cohort of 134,368 individuals, established that NAFLD is associated with increased risks of cardiovascular disease, cancer, and mortality among individuals with T2DM, with hazard ratios showing substantial significance [57].

Moreover, evidence from various ethnic populations, such as the Korean cohort studied by Sung et al. and Kim et al., consistently indicates that NAFLD serves as a strong independent risk factor for CAD [58,59]. These studies revealed a significantly increased prevalence and severity of CAD in patients with NAFLD, emphasizing the broader applicability of these findings. At the same time, CAD was the most prevalent mortality cause among patients with NAFLD in several trials, after all hepatic and non-hepatic cancers were considered [20,60]. Further confirming this association, Mahfood et al.’s 2017 meta-analysis of 25,837 patients across six studies demonstrated that patients with NAFLD had a 77% increased risk of major adverse cardiovascular events (RR: 1.77; 95% CI: 1.26–2.48, *p* < 0.001), including a 126% higher risk of CAD (RR: 2.26; 95% CI: 1.04–4.92, *p* < 0.001), a 109% higher risk of ischemic stroke (RR: 2.09; 95% CI: 1.46–2.98, *p* < 0.001), and a 46% increased risk of CVD mortality (RR: 1.46, 95% CI: 1.31–1.64, *p* < 0.001) [61]. 

The link between NAFLD and cardiovascular outcomes was further demonstrated in Meyersohn et al.’s study, which included 959 patients with liver steatosis [62]. It was found that liver steatosis served as a distinct and independent indicator of MACE in patients, irrespective of conventional CVD risk factors such as obesity or existing CAD. Notably, CVD remains the leading cause of death among individuals with NAFLD, with more than 40% of deaths in this population attributed to cardiovascular events. This epidemiological and pathophysiological link between NAFLD and CVD is critical since CVD is the leading cause of death in the general population, with a rate of >40% in those with NAFLD [63,64]. As NAFLD progresses to more advanced stages, there is an elevated cardiovascular risk, including the development of coronary lesions, increased arterial stiffness, and reduced endothelial function [65,66].

Supporting these findings, Sung et al.’s study of 7371 patients demonstrated an independent association between fatty liver and CAD [48], while Lee et al., in their trial involving 21,335 individuals, found that patients with NAFLD had significantly increased odds of developing CAD compared to those with abdominal obesity alone (OR: 1.286 vs. 1.076) [49]. Another study by Lee et al. in 2018, which included 5121 patients with no prior history of NAFLD or CAD, showed that 38.6% had NAFLD upon ultrasound diagnosis, and these patients had significantly higher odds ratios for developing atherosclerotic plaque (1.18; 95% CI: 1.03–1.35, *p* = 0.016) and non-calcified plaque (1.27; 95% CI: 1.08–1.48, *p* = 0.003) [50]. Lastly, Sinn et al. evaluated 4731 patients with no history of liver or heart disease and found that NAFLD was significantly associated with CAD progression, with the annual rate of CAD progression being 22% in NAFLD patients compared to 17% in non-NAFLD individuals [67].

In summary, recent studies have established a strong association between NAFLD and CAD, demonstrating shared pathophysiological pathways. Research indicates that a significant proportion of CAD patients also have NAFLD, with factors like insulin resistance and increased body mass index playing key roles. Patients with NAFLD show a higher prevalence of CAD and an elevated risk of subclinical atherosclerosis. Meta-analyses have confirmed that NAFLD significantly increases the risk of cardiovascular events, independent of other risk factors such as age, sex, and diabetes. Furthermore, NAFLD patients are at a notably higher risk of major cardiovascular events, including CAD, ischemic stroke, and cardiovascular mortality. The association is consistent across different populations, with studies showing that NAFLD serves as an independent risk factor for CAD. Finally, these findings highlight that NAFLD is a strong predictor of cardiovascular events, underscoring the importance of early detection and management of NAFLD to reduce cardiovascular risks.

Table 1 and Table 2 interpret all the large trials that examine the association of NAFLD with clinical (Table 1) and subclinical (Table 2) CAD.

## 16. Genetic Associations between NAFLD and CAD

The genetic association of NAFLD and CAD is a field of high scientific interest since both conditions seem to be manifestations of interactions between a genetic basis and environmental factors [100]. A recent study by Ren et al. showed an association between genetically predicted NAFLD (defined as chronic elevated alanine aminotransferase levels) and CAD, especially after polymorphisms that are associated with impaired VLDL secretion were excluded [101]. Miao et al. conducted a recent genome-wide association study in 2021, identifying 90 independent NAFLD loci based on common serum and biometric measurements. Through Mendelian randomization analysis, the study revealed the causal effect of NAFLD SNPs on CAD, excluding the reverse relationship [102]. Two genes, sterol regulatory element binding proteins (*SREBP 1-2*), are pivotally implicated in NAFLD. *SREB-1* polymorphisms increase the risk of NAFLD and are linked to the severity of inflammation and necrosis. They may also be associated with CAD through modifications of adhesion molecules to the endothelium. *SREBP-2* polymorphisms influence fatty liver and insulin resistance development. Both *SREBP-1* and *2* single nucleotide polymorphisms (SNPs) predict NAFLD development and EnD [103].

Adiponectin, a hormone derived from adipocytes seem to have properties against atherogenesis, DM and inflammation. Certain alleles of the adiponectin gene are linked to an elevated risk of CAD, while others are associated with a reduced risk, as demonstrated by diverse studies. In patients with NAFLD, adiponectin’s circulating levels are found to be decreased, and some of its polymorphisms, such as *rs266729* has been correlated with NAFLD, its severity, even with the grade of inflammation and necrosis [103]. Apolipoprotein C3 (APOC3), another protein mainly produced in the liver, has been linked to insulin resistance and NAFLD progression in certain populations due to specific gene polymorphisms. Additionally, these polymorphisms are associated with CAD, including cases of acute coronary syndrome, highlighting the role of APOC3 in lipid dysregulation and cardiovascular risk [103,104]. Concerning the use of mRNAs as biomarkers in both conditions, individuals with both NAFLD and CAD, as opposed to those with NAFLD alone, exhibited lower levels of *miR-132* and higher levels of *miR-143* in their blood. This suggests that miRNAs could serve as indicators for detecting and monitoring the progression of these disorders [105]. Exploring the genetic underpinnings of NAFLD and CAD will facilitate the development of gene-based therapeutic approaches, paving the way for more effective precision medicine. This approach aims to identify tailored strategies for each individual to maximize therapeutic benefits while minimizing the risk of adverse reactions.

## 17. Clinical Implications

The clear association between the two entities has been implemented in the latest guidelines of the American Association for the Study of Liver Diseases for the management of NAFLD [106]. More specifically, it is stated that is important for patients with NAFLD to effectively manage CVD risk factors in order to decrease the disease burden and potential risk for MACEs. High awareness is recommended in the modification of high blood pressure, high cholesterol, and hyperglycemia with parallel quitting of smoking [106]. Despite the fact that guidelines support CVD burden in patients with NAFLD, it is critical to underline that individuals with NAFLD should be examined for potential subclinical atherosclerotic disease and, conversely, CVD patients have to be screened for NAFLD. The importance of the preceding strengthens since these two circumstances frequently coexist, and this must ultimately be incorporated into the current guidelines [37].

Furthermore, due to the fact that NAFLD is a common disorder globally, more and more agents targeting steatosis, NASH, and/or fibrosis are being evaluated in phase 2 and phase 3 clinical trials [107]. More importantly, if an agent that is effective in NAFLD is also effective in a subclinical CVD component, this would possibly enhance its benefit in clinical practice, since it can provide survival benefit. Additionally, aside from the beneficial effects on a cardiometabolic variable, another consideration is the treatment indication [37]. One possible therapeutic objective could be to improve NASH, rather than merely NAFLD or any other stage of the disease, since NASH is thought to be a primary driver of its progression [108].

Importantly, therapeutic goals link NAFLD to T2DM. Specifically, in the case of T2DM, the objective is not only to manage blood glucose levels but primarily to reduce the risk of CVD, as the leading cause of mortality in these patients is CVD-related. The T2DM guidelines recommend therapeutic remedies that target both glucose management and CVD risk reduction. Hence, in the case of NAFLD, the primary objective should not just focus on managing liver disease or preventing its progression to malignancy, as CVD is also the main cause of mortality in these individuals. This suggestion seeks to offer a comprehensive recommendation for the guidelines, focusing on the primary causes of mortality, thereby intensifying the burden of NAFLD.

## 18. Optimizing Medical Options

Despite the fact that therapeutic options for treating NAFLD may seem scarce, there is hope that novel and reliable therapies will be accessible in the near future [109]. There are several agents that try to target on the roots of the pathophysiology of NAFLD. First, farnesoid X receptors improve insulin sensitivity, reduce liver gluconeogenesis, protecting from functional impairment caused by potential cholestasis [110]. Obeticholic acid is a novel agent that is generated from bile acids and can bind to farnesoid X receptors to activate that pathway. It is currently being studied in large phase 3 clinical trials in patients with NASH and fibrosis [13,106,111,112].

Furthermore, peroxisome proliferator-activated receptor alpha (PPAR-α) is primarily encountered in the liver and is activated by hypolipidemic fibrates. This molecule regulates the distribution of lipids in the liver by influencing fatty acid transport and depletion through b-oxidation. Additionally, it enhances the levels of HDL cholesterol in the bloodstream and reduces TGs, thereby enhancing plasma lipids. Furthermore, the activation of PPAR-α hinders the expression of inflammatory genes that are stimulated by nuclear factor kB and also reduces the expression of genes associated with the acute-phase response. PPAR-δ has a role in controlling metabolism in both the liver and peripheral tissues. When PPAR-δ agonists are employed, they promote the transport and oxidation of fatty acids, raise HDL levels, and strengthen glucose homeostasis by raising insulin sensitivity and preventing the production of glucose by the liver [113]. Elafibranor is a dual PPAR-α/δ agonist which also improves insulin sensitivity and controls glucose levels and lipid metabolism, while at the same time decreases the inflammatory response of the liver. This drug is also being tested in a large phase 3 clinical study in patients with NASH and fibrosis [114].

Cenicriviroc is a blocker of the C-C chemokine receptor, causing the liver to experience minor inflammation and fibrosis [115]. The migration of macrophages into adipose tissue through the chemokine receptor (CCR)-2 is thought to contribute to the development of insulin resistance and T2DM. The administration of a CCR-2 antagonist led to a slight improvement in glycemic indices when compared to a placebo. The CCR-5 antagonist is anticipated to hinder the migration, stimulation, and reproduction of hepatic stellate cells that produce collagen. In this context, researchers are investigating if this substance can reduce liver fibrosis and alleviate the progression of NAFLD. A randomized, double-blind, phase IIb study revealed promising results, as it improved fibrosis with no worsening of steatohepatitis, compared with placebo [115].

Finally, the MAESTRO-NAFLD study just released the exciting results of resmetirom use in these patients. More specifically, researchers enrolled 996 participants who were randomized to receive placebo or resmetirom at 80 mg or 100 mg [116]. Patients were assessed for the dual primary endpoints of NASH resolution (including a reduction in the NAFLD activity score by ≥2 points) with no worsening of fibrosis and an improvement in fibrosis by at least one stage with no worsening of the NAFLD activity score. NASH resolution with no worsening of fibrosis was achieved in 25.9% and 29.9% of the patients in the 80 mg and 100 mg groups, respectively, vs. 9.7% on placebo. Fibrosis improved by at least one stage with no worsening of the NAFLD activity score in 24.2% and 25.9% of patients in the increasing-dose groups, respectively, compared with 14.2% on placebo. Based on these results, resmetirom has become the first FDA approved agent for the treatment of adults with NASH with moderate to advanced liver fibrosis, to be used along with diet and exercise [116].

In summary, emerging therapies for NAFLD target the disease’s underlying mechanisms, offering new perspectives. FXR agonists enhance insulin sensitivity and reduce liver gluconeogenesis, with some agents in advanced trials for NASH and fibrosis. PPAR agonists improve lipid metabolism, insulin sensitivity, and reduce inflammation, while CCR antagonists have shown potential in decreasing liver fibrosis and inflammation. A recent breakthrough therapy demonstrated significant improvements in NASH resolution and fibrosis, leading to its approval for treating NASH with moderate to advanced fibrosis. These advances represent promising options alongside lifestyle interventions for NAFLD management.

## 19. Conclusions

NAFLD encompasses a broad spectrum of clinical presentations and is strongly linked to CAD through pathophysiological mechanisms that induce EnD. CVD remains the leading cause of mortality among individuals with NAFLD. In light of this, it is imperative to shift from a predominantly NAFLD-centered approach to a more comprehensive framework that incorporates specific evaluation of CVD. Such an approach should not be limited to addressing concurrent risk factors but should ideally encompass the identification of early subclinical CVD manifestations as well. A deeper understanding of the various pathophysiological pathways involved is necessary to fully comprehend the intricate relationship between NAFLD and CVD. This knowledge would pave the way for the emergence of new therapeutic targets and innovative interventions that could potentially benefit individuals affected by both conditions.

## Figures and Tables

**Table 1:** Large trials assessing the connection of NAFLD and symptomatic CAD.

First Author	Year of Publication	Number of Patients	NAFLD and CAD Diagnostic Method	Results
Thévenot [68]	2022	189	Non-invasive fibrosis tests, Fibroscan and coronary angiography	5% of patients with documented CAD had NAFLD.
Hsu [33]	2021	1502	Abdominal ultrasonography and cardiac computed tomography	NAFLD severity is associated with coronary artery atherosclerosis burden, and steatosis severity correlated with the risk of presence of coronary plaques.
Fiorentino [69]	2020	1254	Abdominal ultrasonography and coronary angiography	NAFLD independently associated with an increased risk of having CVD in patients with glucose disturbances.
Langroudi [70]	2018	264	Abdominal ultrasonography and cardiac computed tomography	NAFLD may NOT be associated with atherosclerosis of coronary arteries and its severity in non-diabetic patients.
Sinn [67]	2017	4731	Abdominal ultrasonography and cardiac computed tomography	NAFLD was significantly associated with the development of CAD.
Ishiba [71]	2016	698	Abdominal and cardiac computed tomography	The progression of arteriosclerosis and that of liver fibrosis may be associated in NAFLD patients.
Osawa [72]	2015	414	Abdominal and cardiac computed tomography	Diagnosis of NAFLD may be important when assessing the risk of CAD.
Wong [73]	2015	612	Abdominal ultrasonography and coronary angiography	NAFLD is associated with CAD and need for coronary intervention, but not increased mortality or cardiovascular complications.
Idilman [74]	2015	273	Abdominal and cardiac computed tomography	NAFLD is associated with significant CAD in type 2 diabetic patients.
Puchner [75]	2014	445	Abdominal and cardiac computed tomography	NAFLD is associated with advanced high-risk coronary plaque.
Wong [76]	2011	612	Abdominal ultrasonography and coronary angiography	NAFLD is associated with CAD independently of other metabolic factors.
Sun [77]	2011	542	Abdominal and cardiac computed tomography	NAFLD is associated with high severity of CAD, requiring consciousness in patients with abdominal obesity.
Açikel [78]	2009	355	Abdominal ultrasonography and coronary angiography	NAFLD is associated with the presence of CAD and severity of coronary atherosclerosis.

Abbreviations: CAD: coronary artery disease, NAFLD: non-alcoholic fatty liver disease, CVD: cardiovascular disease.

**Table 2:** Large trials assessing the connection of NAFLD and asymptomatic CAD.

First Author	Year of Publication	Number of Patients	NAFLD and CAD Diagnostic Method	Results
Carter [79]	2022	1726	Abdominal and cardiac computed tomography	Hepatosteatosis was associated with an increased prevalence of CAD.
Ichikawa [80]	2022	1148	Abdominal and cardiac computed tomography	Stable CAD and concurrent evaluation of NAFLD enables more accurate detection of patients at higher risk of MACEs.
Chen [65]	2021	545	Abdominal ultrasonography and cardiac computed tomography	NAFLD was associated with the presence of significant CAD.
Ichikawa [81]	2021	529	Abdominal and cardiac computed tomography	NAFLD in addition to CAD and risk factors, could be useful for identifying diabetic patients at risk of MACEs.
Meyersohn [62]	2021	3756	Abdominal and cardiac computed tomography	Hepatic steatosis is associated with MACEs independently of other cardiovascular risk factors or extent of coronary artery disease. Strategies to reduce steatosis might reduce risk of MACEs.
Saraya [82]	2021	800	Abdominal and cardiac computed tomography	NAFLD is associated with CAD.
Bae [83]	2020	3693	Abdominal ultrasonography and cardiac computed tomography	NAFLD was associated with CAD.
Koo [84]	2020	719	Abdominal and cardiac computed tomography	The association between NAFLD and arterial calcification is mainly mediated by conventional risk factors.
Oni [85]	2019	4123	Abdominal and cardiac computed tomography	NAFLD is associated with carotid atherosclerosis and CAD.
Pais [86]	2019	2617	Abdominal ultrasonography and cardiac computed tomography	Steatosis is associated with carotid and coronary, but not femoral atherosclerosis, and with cardiovascular mortality risk.
Sinn [67]	2019	111,492	Abdominal ultrasonography and cardiac computed tomography	NAFLD was associated with the development of CAD independent of cardiovascular and metabolic risk factors.
Lee [87]	2018	5121	Abdominal ultrasonography and cardiac computed tomography	NAFLD was associated with non-calcified plaque.
Kim [88]	2017	1575	Abdominal and cardiac computed tomography	NAFLD and systemic inflammation increases the risk of CAD over 4 years.
Wu [89]	2017	2345	Abdominal ultrasonography and cardiac computed tomography	NAFLD and CAD associations were significant in female but not in male.
Kim [90]	2016	1472	Abdominal ultrasonography and cardiac computed tomography	Epicardial fat volume and NAFLD are associated with the presence of metabolic syndrome.
Park [91]	2016	1732	Abdominal ultrasonography and cardiac computed tomography	NAFLD was associated with the early development of CAD, but not the progression.
Mellinger [92]	2015	3529	Abdominal and cardiac computed tomography	Association of hepatic steatosis with subclinical CVD.
Al Rifai [93]	2015	3976	Abdominal and cardiac computed tomography	NAFLD is associated with increased inflammation and CAD, independent of traditional risk factors, obesity, and metabolic syndrome.
Lee [94]	2015	10,063	Abdominal ultrasonography and cardiac computed tomography	NAFLD is associated with CAD.
VanWagner [95]	2014	2424	Abdominal and cardiac computed tomography	Obesity attenuates the relationship between NAFLD and subclinical atherosclerosis.
Juarez-Rojas [96]	2013	765	Abdominal and cardiac computed tomography	NAFLD increases the association of metabolic syndrome with type 2 diabetes and subclinical atherosclerosis.
Sung [97]	2013	7371	Abdominal ultrasonography and cardiac computed tomography	Both NAFLD and brachial-ankle pulse wave velocity are associated with CAD.
Kim [59]	2012	4023	Abdominal and cardiac computed tomography	NAFLD patients are at increased risk for CAD.
Sung [98]	2012	3784	Abdominal and cardiac computed tomography	NAFLD and insulin resistance are both associated with CAD.
Lin [99]	2005	2088	Abdominal ultrasonography and digital electrocardiography	Synergistic effect between NAFLD and overweight for developing ischemic heart disease.

Abbreviations: CAD: coronary artery disease, NAFLD: non-alcoholic fatty liver disease, CVD: cardiovascular disease, MACEs: major adverse cardiovascular events.

## Data Availability

Not applicable.
